# A 64-pin Nanowire Surface Fastener Like a Ball Grid Array Applied for Room-temperature Electrical Bonding

**DOI:** 10.1038/s41598-018-37693-2

**Published:** 2019-01-31

**Authors:** Yuhki Toku, Kazuma Ichioka, Yasuyuki Morita, Yang Ju

**Affiliations:** Department of Micro-Nano Mechanical Science and Engineering, Nagoya University, Furo-cho, Chikusa-ku, Nagoya, Aichi 464-8603 Japan

## Abstract

Surface-mount techniques primarily depend on soldering. However, soldering techniques have encountered some challenges in recent years. These challenges include rare metal recycling, thermal problems, and Pb toxicity. We recently developed a metallic nanowire surface fastener (NSF) to resolve the abovementioned problems. This fastener can be used to connect electronic components on a substrate at room temperature using the van der Waals force between each nanowire. This study demonstrates a 64-pin NSF that behaves like a ball grid array (BGA) for application to actual electronic devices. The adhesion strength and electrical properties of the NSF were investigated by adjusting the nanowire parameters, such as diameter, length, density (number per area), preload, and shape. The shape control of the nanowires greatly contributed to the improvement of the properties. A maximum adhesion strength of 16.4 N/cm^2^ was achieved using a bent, hook-like NSF. This strength was 4–5 times the value of the straight NSF. The contact resistivity was 2.98 × 10^−2^ Ω∙cm^2^. The NSF fabricated through the simple template method showed the room temperature bonding ability and adaptability to a highly ordered electrode like the BGA.

## Introduction

High-density electronic packages have increased with the development of nanotechnology. Electric circuits have also become significantly smaller because of the high accuracy micro-/nano-wiring pattern technique. Electronic components, such as the ball grid array (BGA) and the land grid array (LGA), which have many connecting terminals, have facilitated miniaturization and the advancement in the performance of the electronic packages. The BGA and the LGA have an advantage in their small surface mounting area. In addition, the LGA can use high power flow caused by the low contact resistance achieved using pad-shaped terminals instead of solder balls. The physical strength of the LGA is high because of its simple structure^1^. However, some problems exist. Confirming the condition of the solders or reconnecting the components is difficult in the connecting process because almost all terminals are covered by the component body^2^. In addition, the mismatch of the thermal expansion coefficient between the LGA and the substrate, in the case of the LGA, is a problem for future micro-/nano-electronic devices^3,4^. Meanwhile, almost all the surface-mount techniques in the connecting part of the electronic components depend on soldering. However, recoveries and recycles are not easy because of the difficulty in releasing the electronic components from a substrate. Thus, the recovery of such rare metals in the electronic packages incurs significant cost. These problems need to be resolved so that limited natural resources are used. This can be done by developing a new surface-mount technique, which can easily recover the electronic components. Another issue to be addressed is the heat problem. The surface-mount technology with soldering is mainly conducted through a reflow process, where the substrate, on which the solders are printed, is used and heated along with the arranged electronic components on the substrate surface. Accordingly, many types of Pb-free solders have been studied worldwide in an effort to avoid the toxicity of traditional Sn–Pb solders^5–8^. However, Pb-free solders usually have a higher melting temperature than Sn–Pb solders^9^. The melting point of the Sn–Pb solders is 183 °C, while that of Pb-free solders is 200 °C. Thus, the temperature in the reflow process must be raised to over 240 °C, which includes a margin of approximately 40 °C^9–11^. Meanwhile, the electronic components have a low thermal strength and would not be suitable for the reflow process. Moreover, a high current density recently caused thermal cracks^12–16^ in the connecting part of the electric circuits, which are mainly caused by power devices^10,17^ because of the low thermal conductivity of the connecting part. Therefore, the electronic components lead to failure because the heat is confined. Heat confinement is also a problem to be solved in the surface-mount technique.

The biomimetic adhesive technique, which has been developed by using nanowire or nanotube arrays, is a candidate for solving the above problems^18–25^. We recently proposed a nanowire surface fastener (NSF)^26–29^, which applies a conductive method that utilizes the van der Waals force between each nanowire to connect to the electronic components. Metallic nanowires, such as Cu and Au, contribute to the high electrical and thermal conductivities. The NSF can be repeatedly used. Moreover, the recycle, repair, or disposal of the electronic components is easy compared with soldering. Ju *et al*. developed an NSF composed of an Au nanowire array for which mechanical and electrical bonding was realized at room temperature^26^. The adhesion strength was greater than 5 N/cm^2^ and the parasitic resistance was around 2 Ω (contact resistivity: 6.28 × 10^−2^ Ω·cm^2^)^26^. Wang *et al*. fabricated a copper nanowire array NSF^27^. An adhesion strength of 8.17 N/cm^2^ and a contact resistivity of 0.69 × 10^−2^ Ω·cm^2^ were achieved. In addition, Wang *et al*. fabricated a copper/parylene core/shell NSF to improve the mechanical strength^28^. This NSF exhibited an adhesion strength of ~25 N/cm^2^ and a contact resistivity of ~4.2 × 10^−2^ Ω·cm^2^. Meanwhile, a new model was developed to analyze the van der Waals forces between the core/shell NWs^28^. Wang *et al*. also demonstrated a new copper/polystyrene core/shell NSF, which showed a higher adhesion strength (~44.42 N/cm^2^) and a much lower contact resistivity (~0.75 × 10^−2^ Ω·cm^2^)^29^. However, all the studies were conducted based on a 4-pattern (4-pin) nanowire array, which is still far from the actual surface mount application. Moreover, the adhesion strengths of those NSFs were clearly different in shear and normal directions (i.e. the normal adhesion strength is much lower than the shear one) due to all the nanowires being straight.

In this study, we report the fabrication of a BGA-like 64-pin Cu NSF, used for the surface mounting of electronic components. The adhesion strength and contact resistivity were investigated by adjusting the nanowire parameters, including the diameter, length, wire density, preload, and shape. Especially, the hooked nanowire array which has a structure like a Velcro was firstly introduced in this study in order to investigate the effect of shape control on the properties of NSF, as well as the homogeneity of the adhesion strength in shear and normal directions.

## Experiment

**Figure 1:**
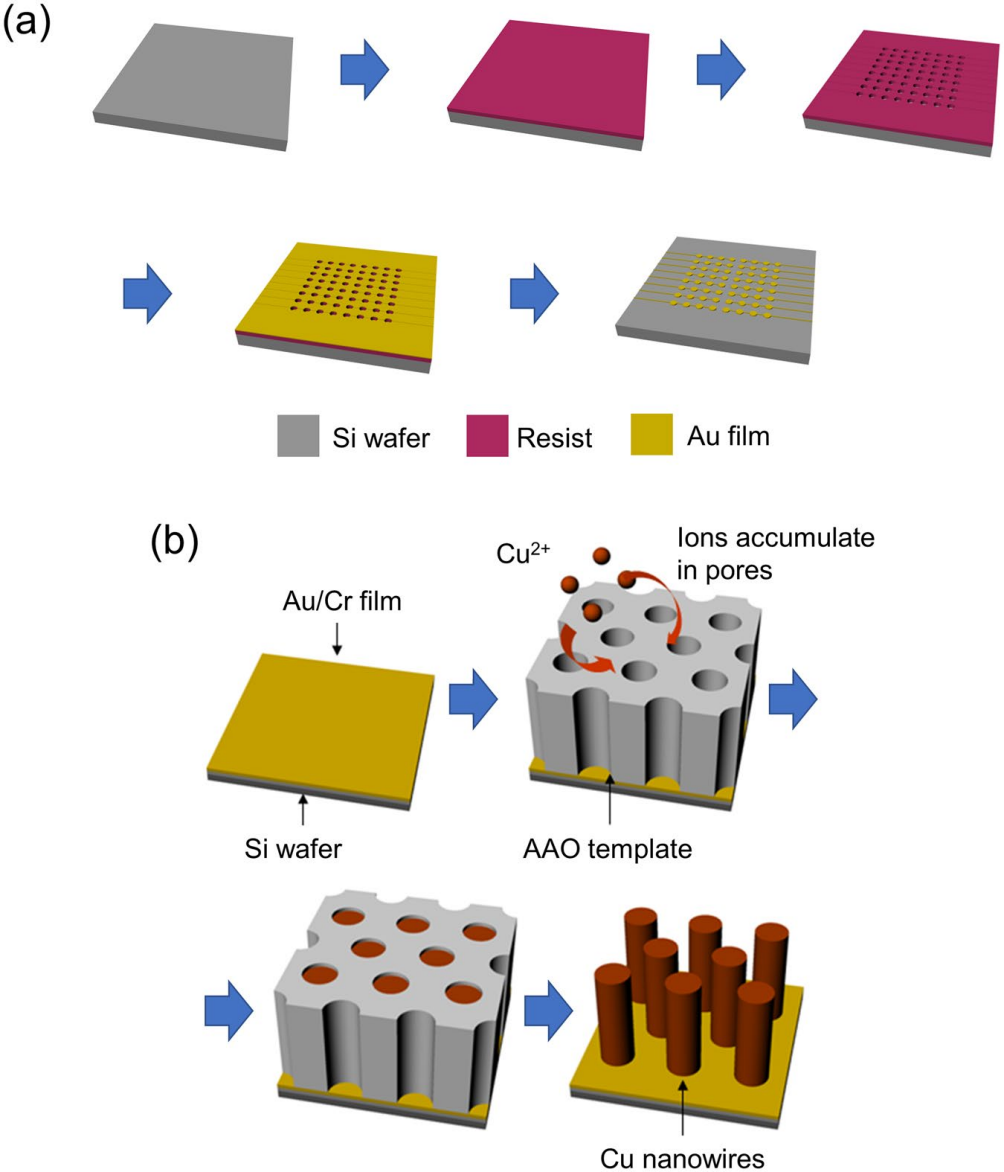
(**a**) Procedure followed to prepare 64 pad electrodes using the lift-off technique. (**b**) Template method procedure using the AAO template. The nanowires are composed of electrodeposited Cu.

Figure 1(a) presents the preparation procedure of the 64-pin electrode pattern for the NSF fabrication where a MEMS process was introduced. First, an Si substrate was cleaned by an ultrasonic cleaning process using acetone, ethanol, and pure water. The ultrasonic cleaning lasted for 5 min, and was 42 kHz. Subsequently, an electric circuit was prepared on the substrate using the lift-off technique. A photoresist was prepared for 30 s on the substrate using a spin coater with 2000 rpm. The Cr and Au films were then sputtered on the patterned photoresist after the lithography process. Finally, a lift-off was conducted by ultrasonic cleaning etching with acetone. We then obtained the circuit of a 64-pad pattern, which was confirmed by the scanning electron microscope observation (SEM; JEOL, FESEM JSM-7000FK). The diameter of each electrode pad was 0.5 mm.

The NSFs were fabricated using a template method, which can fabricate highly ordered metallic nanowires. Figure 1(b) illustrates the experimental procedure of the template method. The template method mainly proceeded according to the following procedure: First, the template—a nanoporous membrane—was set on the electrode. Second, the pores were filled with metallic atoms through the electrodeposition technique. Last, the template was etched, and the highly ordered metallic structures, with the same shape as the pores, were obtained. The experimental setup of the template method is shown in Fig. S1 of the supplementary information, with such details as how to fix the template. Two types of anodic aluminum oxide (AAO) template were used in the electrodeposition process (i.e., Whatman Anodisc13 (diameter: 13 mm; thickness: 0.06 mm; pore diameter: 20 nm; distance between each pore: 40 nm; and pore density: 10^11^ cm^−2^) and Synkera UniKera (diameter: 13 mm; thickness: 0.05 mm; pore diameter: 80 nm; distance between each pore: 240 nm; pore density: 2 × 10^9^ cm^−2^)). The template and the substrate were fixed together by self-made acrylic jigs. The substrate with the template was soaked in electrolyte for 3 h to completely fill the pores of the template. An amount of 0.4-M copper (II) sulfate pentahydrate, which was controlled to a pH of 2 by sulfuric acid, was prepared as an electrolyte solution. The applied current condition was controlled to be approximately 16 mA/cm^2^. The nanowire length was controllable by changing the electrodeposition duration. Template etching was conducted in a 2-M NaOH solution. The etching time was 6 h. After that, the sample was cleaned with deionized water. The sample was then dried by natural or supercritical drying. The structure and element analysis of the NSF was conducted by transmission electron microscopy (TEM; JEOL, JEM-1000K RS).

**Figure 2:**
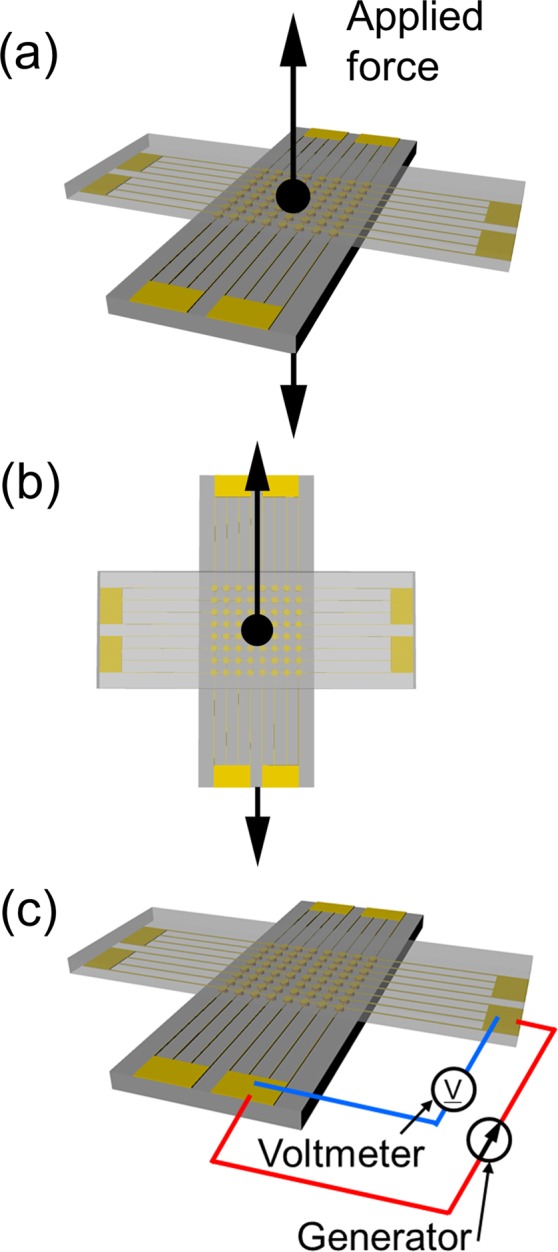
Mechanical and electrical test setup. (**a**,**b**) Show the tensile test applied to the normal and shear directions, respectively. The arrows indicate a tensile direction. (**c**) Current test setup.

The Cu NSF consisted of two Cu nanowire arrays connected to face each other (Fig. 2). The fabricated NSFs were mechanically and electronically evaluated. The mechanical property was investigated by a tensile test applied to the normal and shear directions (Fig. 2(a,b), respectively). The NSF was set on the tensile tester. Another side was connected to a load cell with a rope. The electric property was then investigated by a current test (Fig. 2(c)). The electrical resistance of the Cu NSF was measured by a traditional four-point probe method^30^. A current source (263 Calibrator/source, KEITHLEY) was used to apply a constant current through the two outer terminals, and a nanovoltmeter (2182A Nanovoltmeter, KEITHLEY) was used to measure the voltage through the two inner terminals. Therefore, the constant resistance between the terminal and the Au film can be neglected. The contrast of the electrical properties was more generally made by multiplying the contact resistivity expressed by the measured resistance by the contact area of the NSF (unit: Ω∙cm^2^)^31–33^. In the mechanical test (Fig. 2(a,b)), all the NSFs located on the 64 pads were used for the experiment, while 16 pads were utilized for the current test (Fig. 2(c)). Thus, their active contact areas in these experiments were approximately 12.56 mm^2^ and 3.14 mm^2^, respectively.

We used several conditions for the NSF in these tests, including the nanowire length, diameter, preload for connecting, and the nanowire shape. The investigated conditions were as follows: lengths of 10, 15, 20, and 30 μm; diameters of 20 nm and 80 nm; nanowire shape of hooked or straight; and preloads of 4.9, 9.8, and 19.6 N (39, 78, and 156 N/cm^2^). The tests were conducted three times.

First, we investigated the influence of the drying method and the template type on the NSF properties. The NSF in this experiment was examined under the conditions of 9.8-N preload and 15-μm nanowire length. The influence of the nanowire length and the preload on the NSF properties was then investigated using the determined template and drying method. We also investigated the influence of the nanowire shape on the NSF properties using the pre-determined conditions (template, drying method, nanowire length, and preload). Accordingly, the poly-carbonate film was used to uniformly deform the nanowires. The tops of the nanowires were permanently bent by applying a shear force with the film (Fig. S2(a) in the supplementary information). This process caused the hooked nanowires (e.g., Velcro) to improve the adhesion strength of the NSF. The hooked nanowire arrays were connected to face each hook direction under a preload. In addition, the hooked nanowire array on each electrode pad was bent in several directions to strongly lead them to any direction (Fig. S2(b) in the supplementary information).

## Results and Discussion

**Figure 3:**
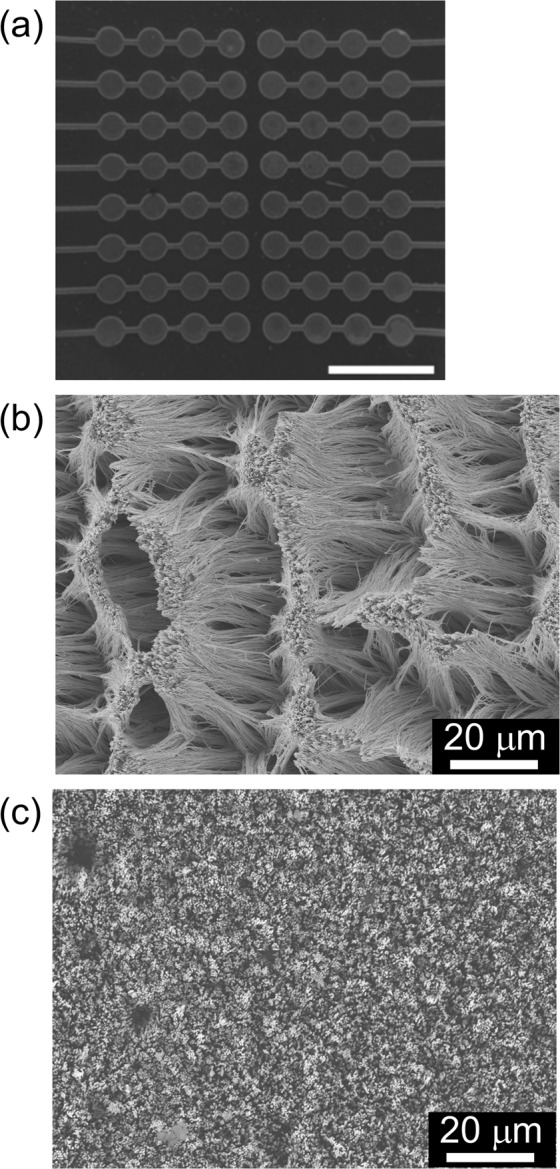
(**a**) SEM image of the final electrode state (scale bar = 2 mm) and (**b**,**c**) the NSF top views. The drying conditions are as follows: (**b**) natural drying and (**c**) supercritical drying.

The relationship between the nanowire length and electrodeposition time was investigated accordingly. The early electrodeposition stage (up to 1 h) exhibited no nanowire growth because the electrodeposited copper was deposited on the slight gap between the template and the substrate. The pores of the template were then filled at an electrodeposition rate of approximately 10 μm/h, which was the nanowire growth rate as shown in Fig. S3 of the supplementary information. We confirmed the fabricated 64-pin NSF using SEM (Fig. 3(a)), where each electrode pad was 0.5 mm in diameter. The difference between natural and supercritical drying was observed as the difference caused by the agglomeration behavior (Fig. 3(b,c)). The NSF was agglomerated by the water surface tension (Fig. 3(b)). Meanwhile, the NSF was not agglomerated when supercritical drying was applied (Fig. 3(b)). We also confirmed the uniformity of the nanowire length from these observations.

**Figure 4:**
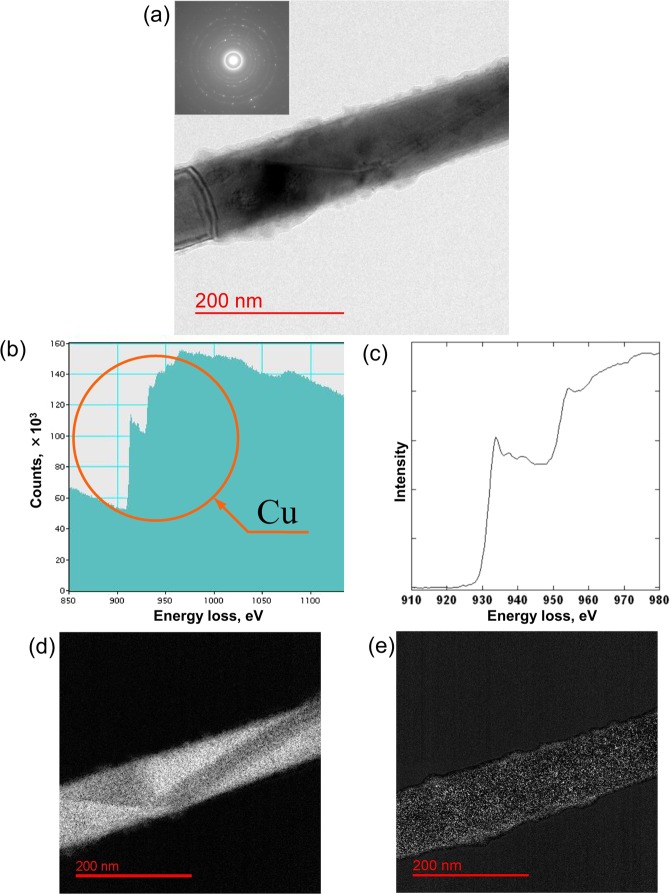
(**a**) TEM image of a Cu nanowire. The inset indicates the diffraction patterns around the center of the nanowire. (**b**,**c**) Results of the electron energy loss spectroscopy analysis around the center of the Cu nanowire: (**b**) measured from the nanowire and (**c**) measured from the pure Cu sample. (**d**) Cu and (**e**) O_2_ mapping around the center of the nanowire.

We analyzed the individual Cu nanowires to confirm the Cu nanowire structure fabricated by the template method using TEM (Fig. 4(a)). The inset of Fig. 4(a) shows the diffraction pattern around the center of the nanowire. The diffraction pattern confirmed that the nanowires exhibited a polycrystalline structure. Figure 4(b) presents the results of the electron energy loss spectroscopic analysis around the center of the nanowire. The nanowires almost agreed with the reference sample of Cu (purity: over 99.9%) (Fig. 4(c)). Figure 4(d,e) illustrate the Cu and O_2_ mapping, respectively. These results indicated that the oxidization was distributed throughout the entire nanowire surface but appeared like tiny dots. Thus, the Cu nanowire probably did not conspicuously oxidize. Thus, the influence of oxidization could be neglected in all the experiments.

**Figure 5:**
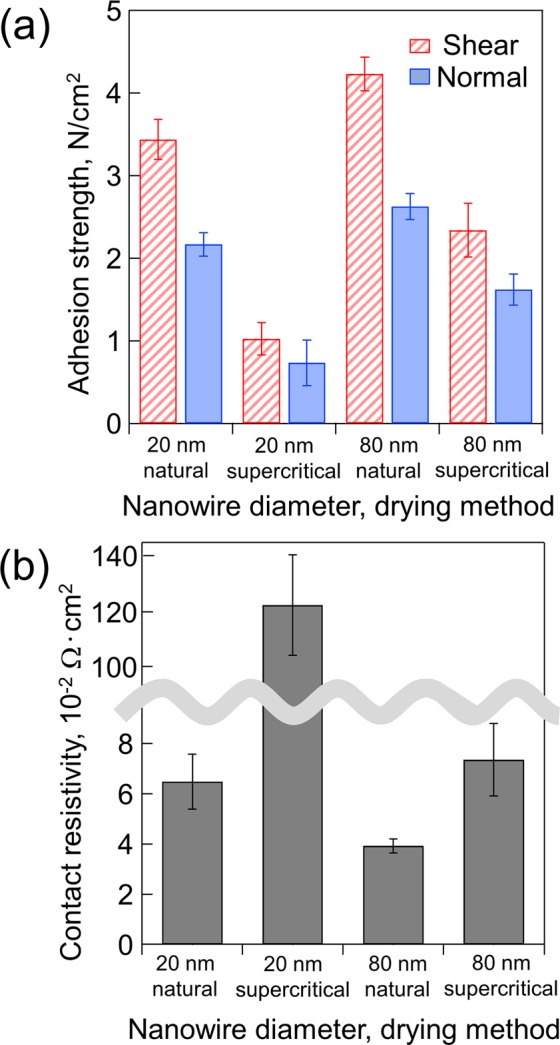
Comparison of the properties of the NSF fabricated by different conditions: (**a**) adhesion strength and (**b**) contact resistivity.

The adhesion strength and contact resistivity were different according to the templates and drying methods (Fig. 5). The results indicated that the 80-nm template and natural drying were the best conditions for NSF fabrication. The adhesion strengths in the shear and normal directions were approximately 4.3 N/cm^2^ and 2.7 N/cm^2^, respectively. The contact resistivity was approximately 3.71 × 10^−2^ Ω∙cm^2^. These results were achieved because the nanowires were not agglomerated in the case of supercritical drying; thus, a few vacant spaces were found in the connecting process. Therefore, the nanowires did not have a sufficient contact area to connect with each other. On the other hand, the nanowires were agglomerated by surface interaction forces in the case of natural drying, which produced a space for the nanowires to connect with each other, thereby resulting in their ability to make contact because of the increase in the contact areas. Figure S4 shows an example of the cross-sectional views of the NSFs interconnection part. More detailed observations and discussion of the interconnection, monitoring such details as the connection behavior changing with increase of the preload, was reported in our previous study^27^. The relationship between the adhesion strength and nanowire pitch is also shown in Fig. S5 in the supplementary information. As can be seen from Fig. S5, in the case of supercritical drying, the adhesion strength is increased with increase in nanowire pitch, which indicates that the pitch of the nanowires will also significantly affect the adhesion strength of the NSF. We used the 80-nm template and natural drying conditions for the following experiments based on this experimental result.

**Figure 6:**
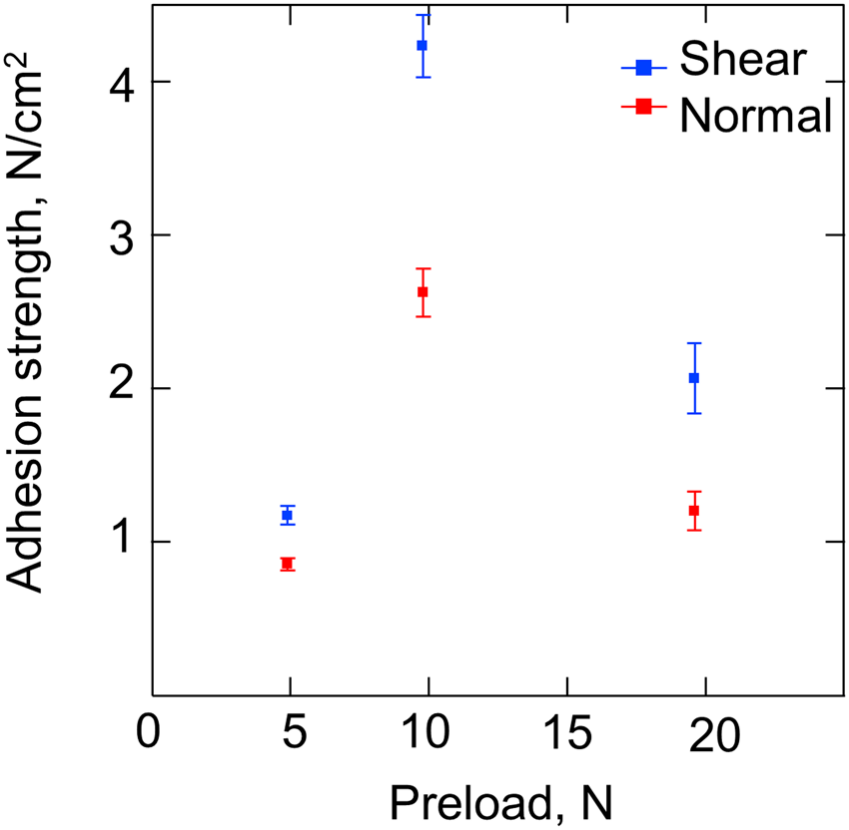
Adhesion strength of the shear and normal directions as a function of the preload.

**Figure 7:**
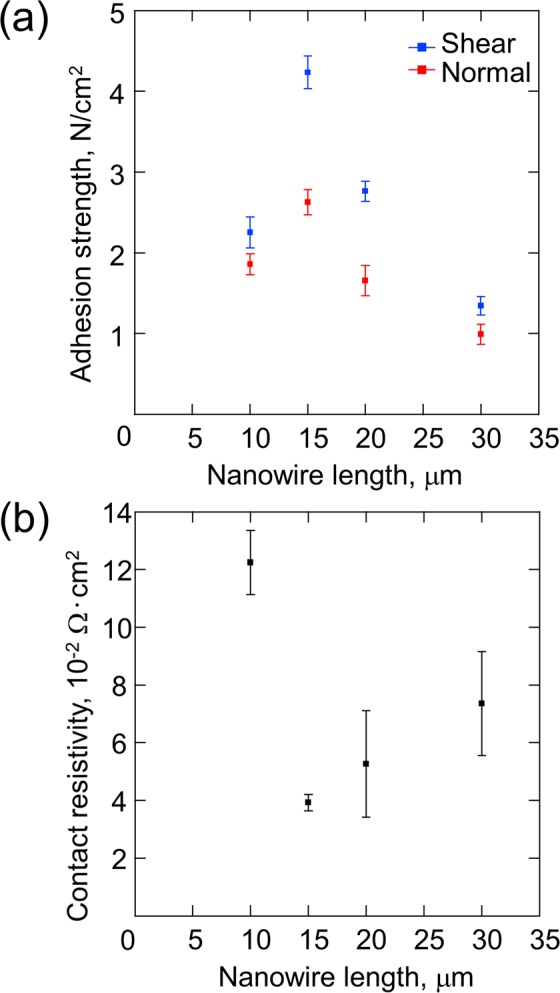
(**a**) Relationship between the adhesion strength and the nanowire length. The maximum adhesion strength appears under the condition that the nanowire length is 15 μm. (**b**) Relationship between the contact resistivity and the nanowire length. The minimum contact resistivity appears under the condition that the nanowire length is 15 μm.

The 9.8-N preload resulted in the maximum adhesion strength along the shear and normal directions, with a 15-μm nanowire length (Fig. 6). This result was also observed in the case of the other nanowire lengths, as shown in Fig. S6 of the supplementary information. Figure 7(a) shows the relationship between the adhesion strength and the nanowire length under the 9.8-N preload condition. The nanowire length, which contributed to the maximum adhesion strength, was 15 μm (Fig. 7(a)). Figure 7(b) presents the result of the current test. The lowest contact resistivity was observed under the conditions of 9.8-N preload and 15-μm nanowire length. The tensile strength and the contact resistivity depended on the contact area of the NSF. Thus, the maximum contact area probably occurred under the conditions of 9.8-N preload and 15-μm nanowire length.

**Figure 8:**
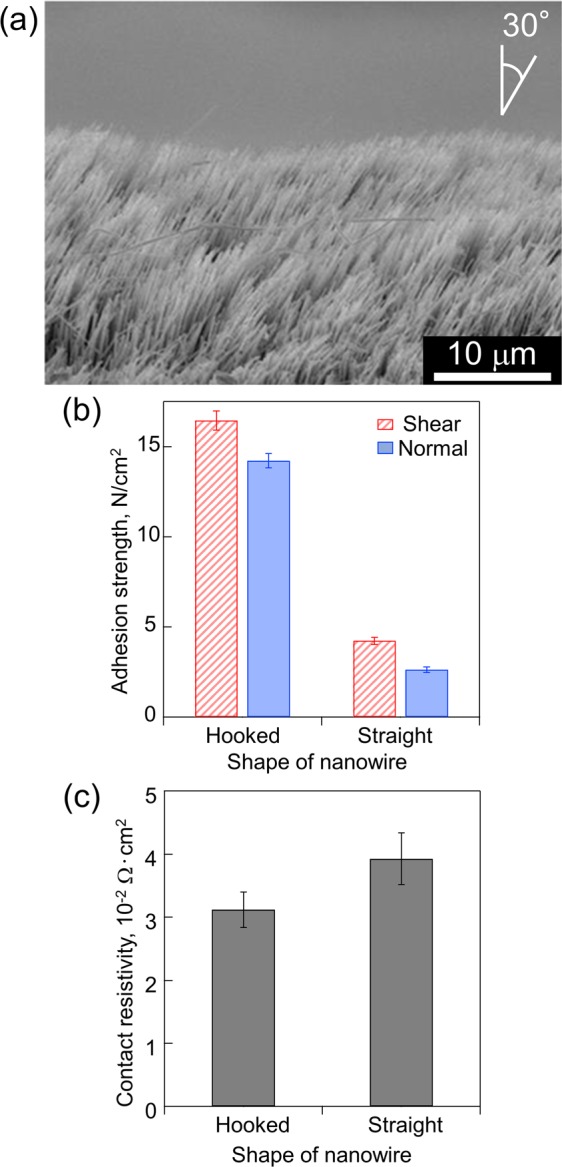
(**a**) Oblique perspective figure of the hooked NSF. (**b**) Adhesion strength and (**c**) contact resistivity of the hooked NSF compared with those of the straight NSF.

We conducted an additional experiment of the tensile and current tests with a hooked NSF to improve the mechanical adhesion strength. This experiment used the best conditions of adhesion strength mentioned earlier (nanowire length: 15 μm; preload: 9.8 N). Figure 8(a) shows the hooked NSF, which was bent 30° relative to the vertical direction. Similar shapes were found in each pad. Figure 8(b,c) illustrate the adhesion strength and contact resistivity of the hooked NSF in comparison with those of the straight NSF. The hooked NSF has adhesion strengths of 16.4 N/cm^2^ and 14.3 N/cm^2^ along the shear and normal directions, respectively. In addition, its contact resistivity was 2.98 × 10^−2^ Ω∙cm^2^. The hooked shape produced an adhesion strength of 4 to 5.5 times more than that of the straight NSF. The adhesion strengths in the shear and normal directions showed close values, whereas the straight NSF had a low adhesion strength in the normal direction. This result was obtained because the hooked nanowires worked as mechanical connectors. Thus, the normal adhesion strength, which originally depended on the surface interaction force, changed to mechanical strength connected by the hooked shape of the nanowires. Meanwhile, their contact resistivity was not sufficiently large to distinguish the hooked NSF from the straight NSF. These results indicated that the contact area did not dramatically change, regardless of whether the nanowires had a bending shape or not. Pb-free solder exhibits a shear adhesion strength of 1000–4000 N/cm^2^ and a contact resistivity of 1.56 × 10^−6^–6.25 × 10^−6^ Ω·cm^2^, respectively^34^. Compared to them, the adhesion strength and contact resistivity of the NSF obtained in this study are still not able to meet the requirements of practical application. However, it should be mentioned that the high density of nanowires prevents an area of contact between each nanowire. Therefore, a low-density nanowire array, i.e., enough pitch in between the nanowires, would be able to increase the adhesion strength sufficiently. Reportedly, the theoretical shear adhesion strength of Cu NSF can reach up to 499.2 N/cm^2^ when each nanowire is in contact with each other and the contact length is 300 nm^27^. In addition, the contact resistivity can also be decreased with increase in contact area of the nanowires. Regarding the reusability of the NSF, there was no significant change in the adhesion strength within 3–5 cycles of disassembly and re-assembly, which was reported in our previous study^26^.

## Conclusions

We demonstrated a 64-pin BGA-like nanowire surface fastener (NSF). The mechanical and electrical properties of the NSF were also investigated. In addition, the hooked NSF was fabricated to improve the tensile strength. We successfully increased the tensile strength five-fold, compared with the straight NSF. The hooked NSF had a shear adhesion strength of 16.4 N/cm^2^ and a normal adhesion strength of 14.3 N/cm^2^. We also confirmed that the contact resistivity was not very different even if the nanowires had a deformed hook-like shape. These results indicated that the mechanical connection in the NSF connection had increased. Our Cu NSF, which was fabricated using a simple template method, showed the room temperature bonding ability and the adaptability required for a highly ordered electrode, like the BGA. The NSF is also expected to become a low-cost surface-mount technique in the future because the reflow process is not necessary. In addition, the Cu NSF is expected to improve the thermal properties of the future surface-mount technique based on the high thermal conductivity of Cu.

## Supplementary information

Supplementary information
