# Supplementary material for: A 64-pin Nanowire Surface Fastener Like a Ball Grid Array Applied for Room-temperature Electrical Bonding
Source: Sci Rep. 2019 Jan 31;9:1095. doi: 10.1038/s41598-018-37693-2 (PMC6355887; doi:10.1038/s41598-018-37693-2)
Supplement: Supplementary file 1 — Supplementary information [file 41598_2018_37693_MOESM1_ESM.docx]

**Supplementary Information**

A 64-pin Nanowire Surface Fastener Like a Ball Grid Array Applied for Room-temperature Electrical Bonding

Yuhki TOKU, Kazuma ICHIOKA, Yasuyuki MORITA, and Yang JU^*^

Department of Micro-Nano Mechanical Science and Engineering, Nagoya University, Furo-cho, Chikusa-ku, Nagoya 464-8603, Japan

**Figure S1.** Schematics of the template method. (a) Preparation of the patterned electrode. (b) Fix the template on the electrode by jigs. (c) Before electrodeposition. (d) After electrodeposition. (e) Etching the template.


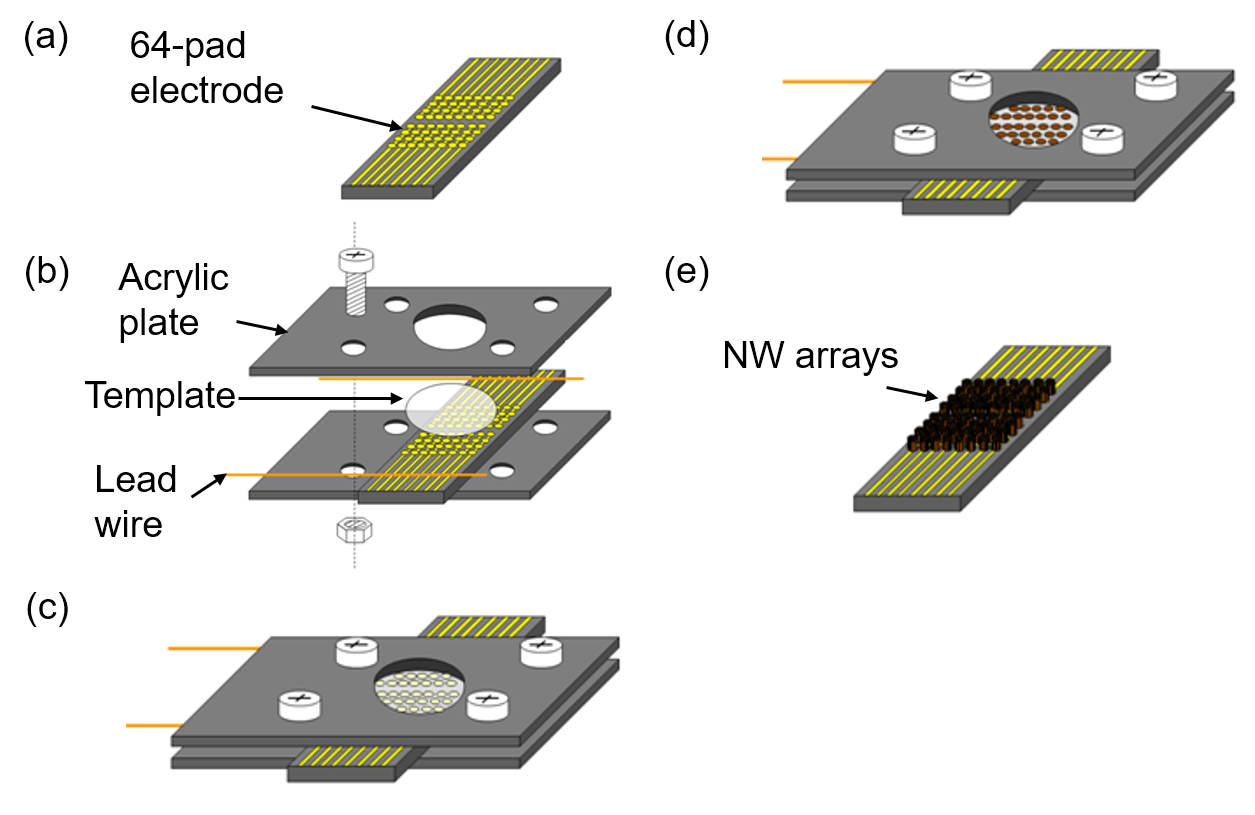


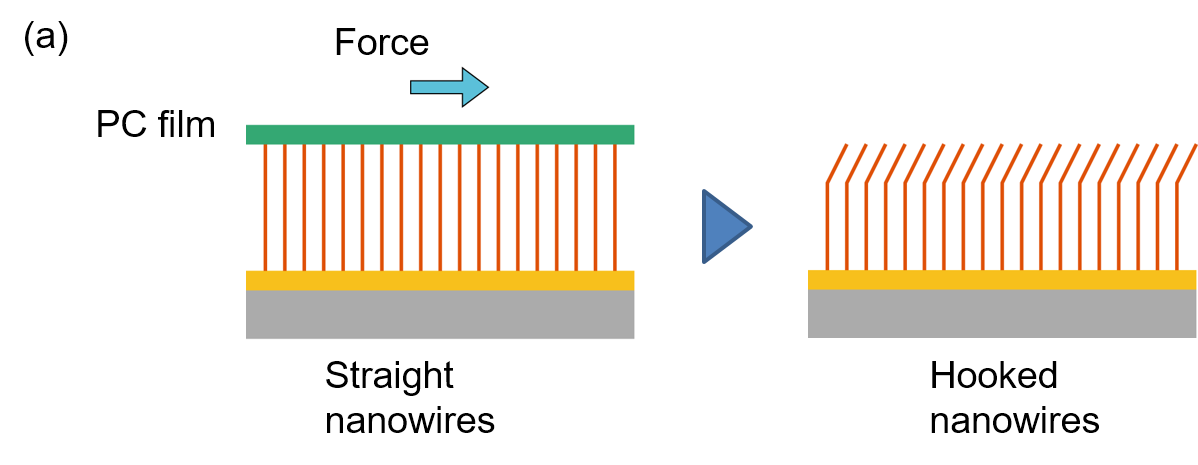


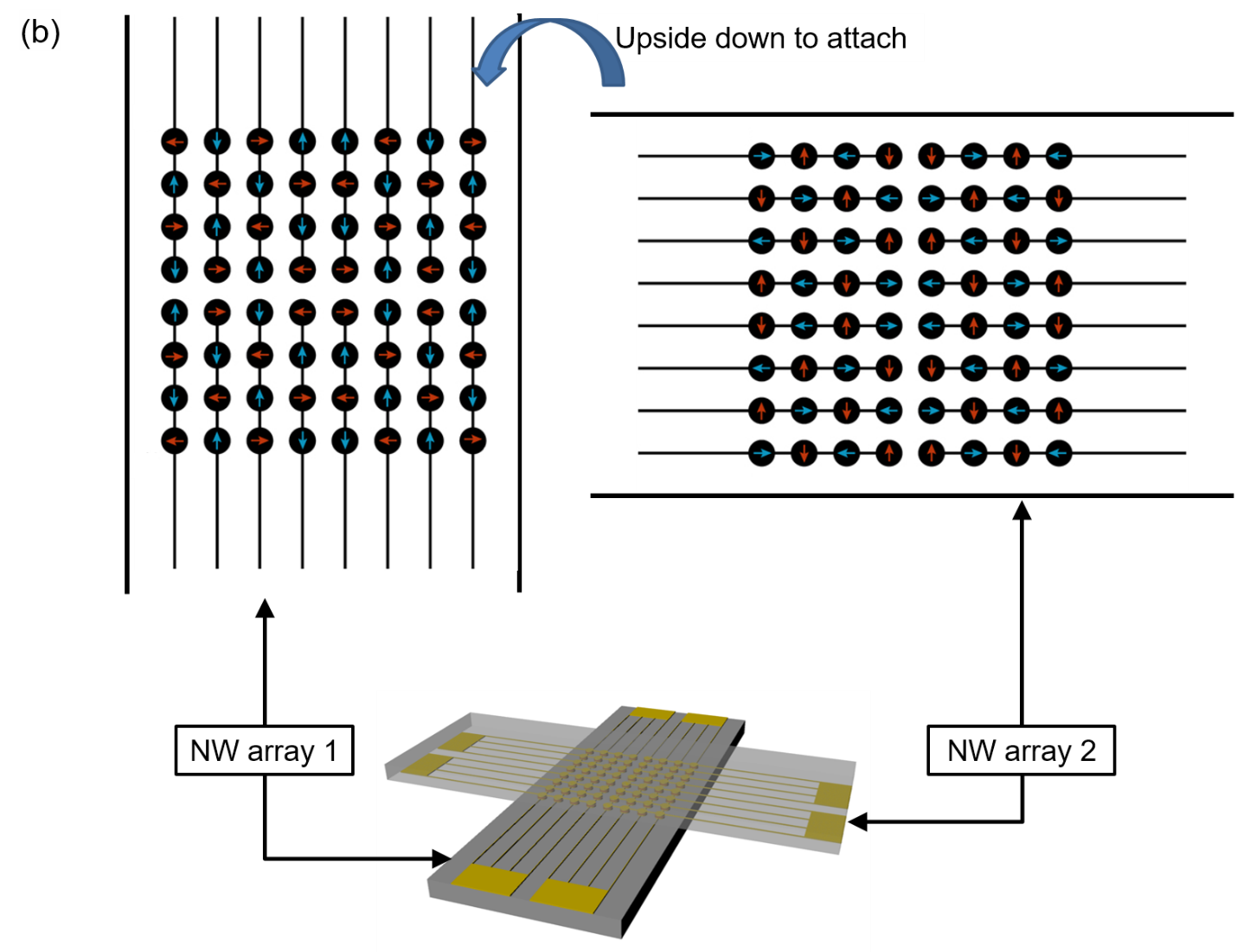


**Figure S2.** (a) The top of nanowires was bent by applying the shear force with the polycarbonate (PC) film. (b) Direction of hook in 64 pads. The hooked nanowire arrays were connected to face each hook direction.


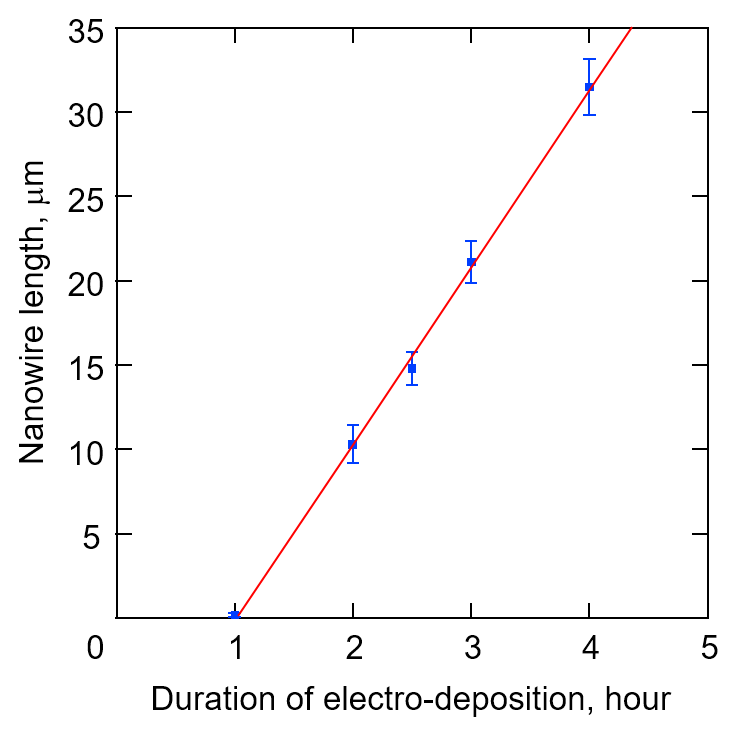


**Figure S3.** The nanowire growth rate during the electrodeposition. The early electrodeposition stage (until 1 h) exhibited no nanowire growth because the electrodeposited copper was deposited on the slight gap between the template and the substrate.


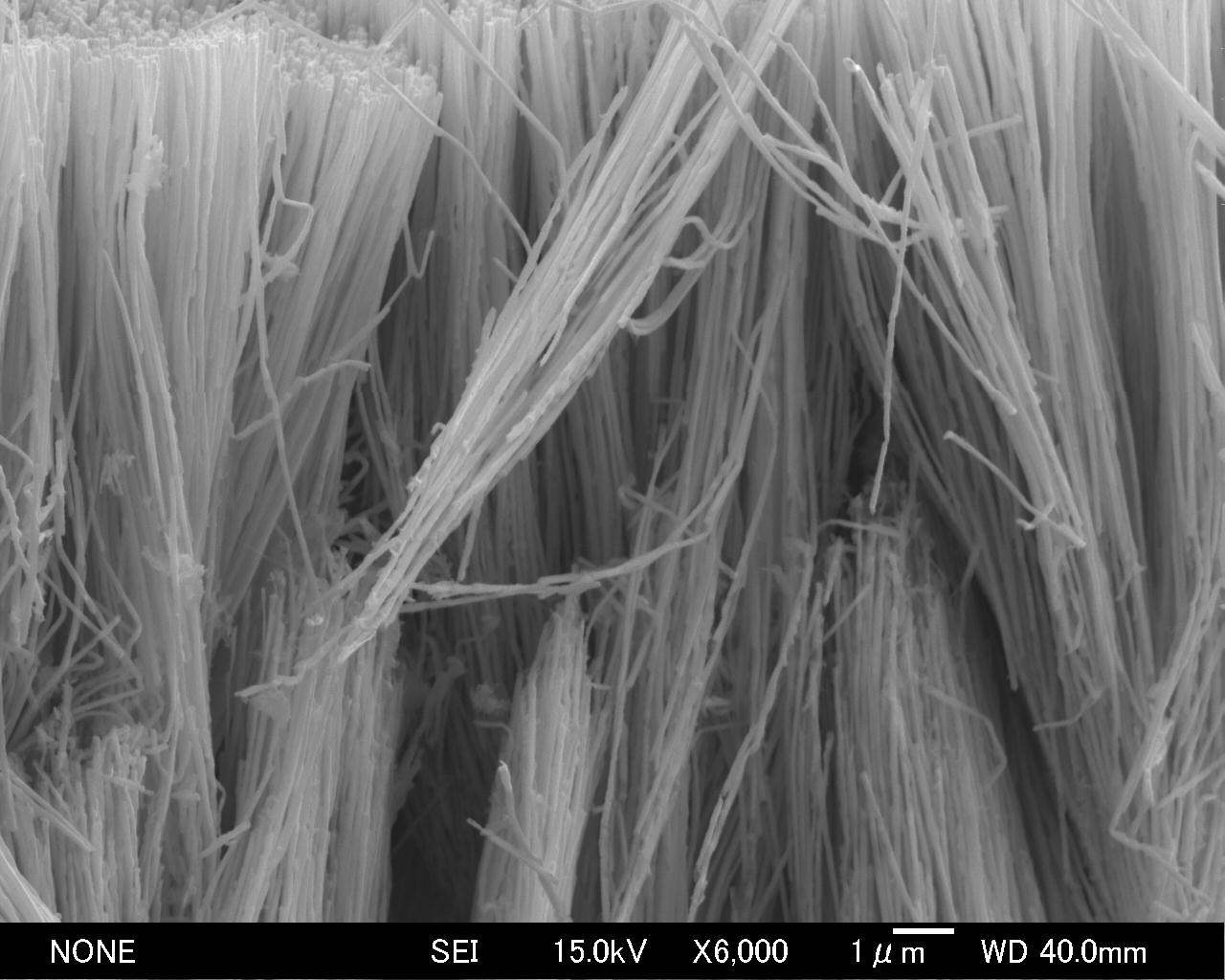


**Figure S4.** SEM image of the cross-sectional view of the NSFs interconnection part assembled under the conditions of nanowire diameter of 80 nm, nanowire length of 10 μm, preload of 9.8 N, and normal drying, respectively.


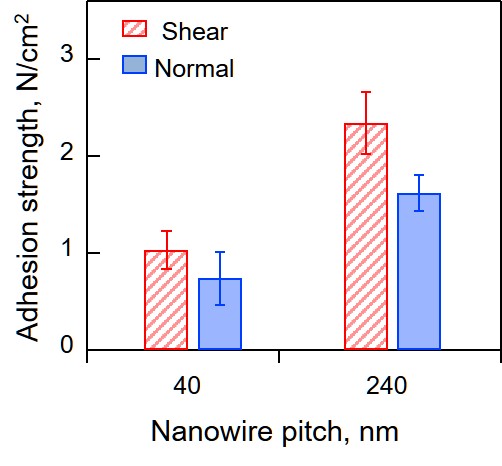


**Figure S5.** The relationship between the adhesion strength and nanowire pitch under supercritical drying condition.

.


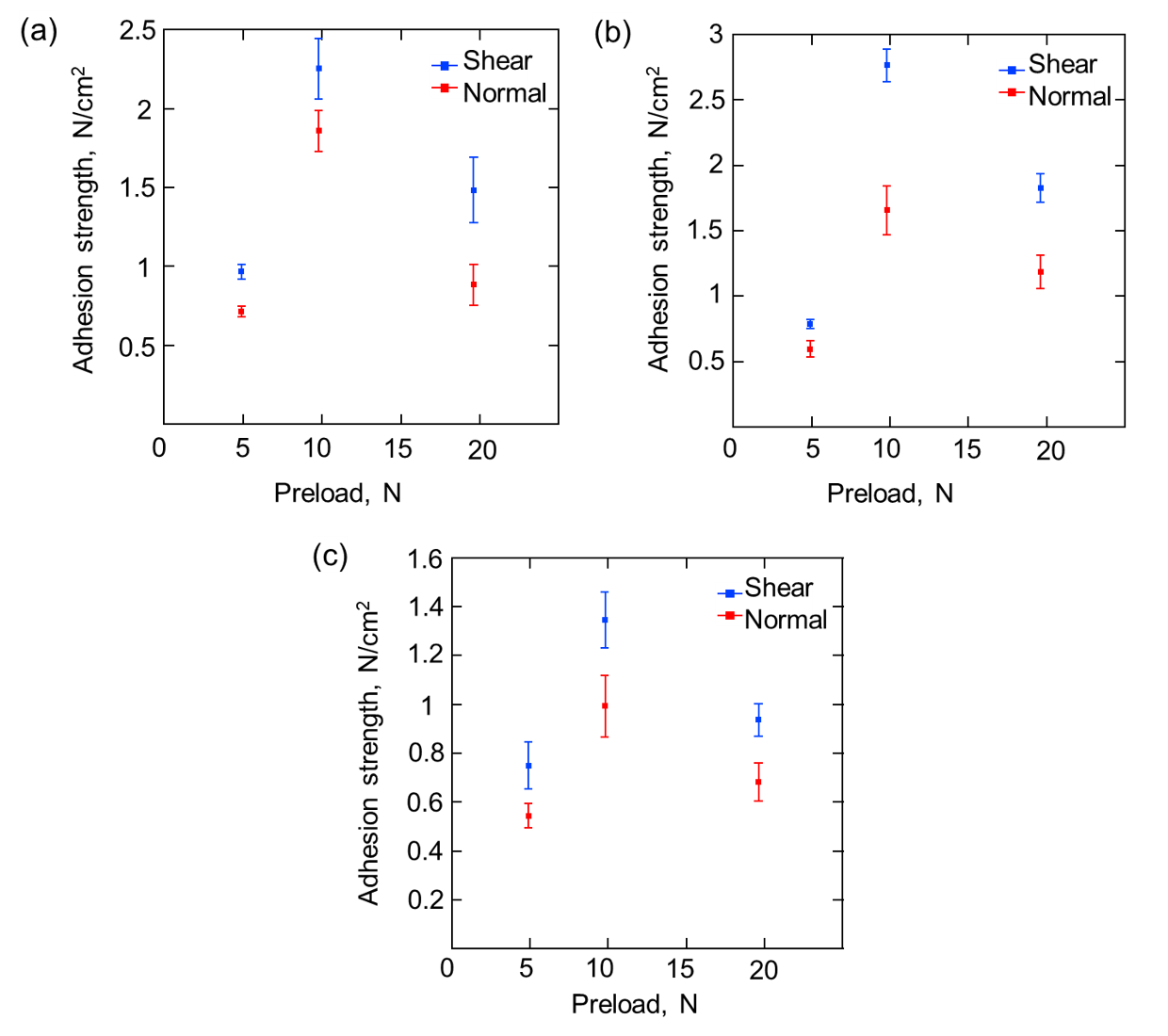


**Figure S6.** Adhesion strength of the shear and normal directions versus the preload for nanowire length of (a) 10 μm, (b) 20 μm, and (c) 30 μm.
